# Integrating musculoskeletal ultrasound as a shared decision-making tool in hemophilia care: observations from a 3-year study

**DOI:** 10.1016/j.rpth.2024.102511

**Published:** 2024-07-14

**Authors:** Azusa Nagao, Satoko Orita, Katsuyuki Fukutake, Hideyuki Takedani

**Affiliations:** 1 Department of Blood Coagulation, Ogikubo Hospital, Tokyo, Japan; 2 Department of Rehabilitation, Ogikubo Hospital, Tokyo, Japan; 3 Department of Laboratory Medicine, Tokyo Medical University, Tokyo, Japan; 4 Department of Joint Surgery, Hospital of the Institute of Medical Science, The University of Tokyo, Tokyo, Japan; 5 Department of Rehabilitation, NHO Tsuruga Medical Center, Fukui, Japan

**Keywords:** decision making, hemophilia, musculoskeletal ultrasound (MSKUS), subclinical, synovitis

## Abstract

**Background:**

Hemophilia significantly impacts joint health, necessitating innovative strategies for early detection and management of joint damage.

**Objectives:**

This study assessed the impact of incorporating musculoskeletal ultrasound (MSKUS) into shared decision-making processes on prophylaxis regimens for patients with hemophilia over a 3-year period.

**Methods:**

The “Joint Damage Monitoring by Ultrasonography in Patients with Hemophilia in Japan” study was a long-term prospective observational study conducted at Ogikubo Hospital, Tokyo, Japan. It enrolled 174 participants with moderate-to-severe hemophilia A or B. Participants underwent 6 monthly MSKUS evaluations, generating 3582 images from 682 joints; the findings guided adjustments of prophylaxis.

**Results:**

Over the 3-year period, 69.3% of participants adjusted their prophylaxis regimen at least once. Adherence, defined as the ratio of the prescribed to the actual frequency of prophylaxis administration as agreed upon by physicians and patients, was high at the beginning of the study, with an average of 91.6%, and remained high after 3 years at 94.7%. The HEAD-US scores for elbows, knees, and ankles significantly improved (all *P* < .0001). The spontaneous annual joint bleeding rate and Hemophilia Joint Health Scores also significantly improved (*P* = .001 and *P* = .004, respectively). Synovitis detection decreased from 12.9% to 1.6%, with the majority of identified synovitis being subclinical (11.7%) and not associated with bleeding events in the 6 months preceding detection.

**Conclusion:**

Integrating MSKUS into hemophilia care as a shared decision-making tool significantly facilitates the early detection of joint damage and supports personalized prophylaxis adjustments, markedly improving patient outcomes.

## Essentials

• Hemophilia affects joints; new ultrasound method aids in care.
• J-DaUPHIN study was conducted in Japan; 174 participants were involved.
• Prophylaxis adjustments were made based on ultrasound findings.
• Improved joint health was observed; shared decision making was found to be crucial.

## 1 Introduction

Individuals with hemophilia are at risk of joint bleeding, which, if recurrent, can escalate to arthropathy, thus significantly deteriorating mobility and quality of life. Despite significant progress in hemophilia treatments, prophylaxis is not always successful in arresting arthropathy progression among all individuals with hemophilia [1]. Moreover, the annual bleeding rate (ABR), an indicator based on patient self-reporting, cannot predict the joint outcomes accurately. It has been postulated that insidious, subclinical bleeding, which is potentially imperceptible to the patients themselves, might exert consequential impacts on the joint prognosis even in patients receiving appropriate prophylaxis [2]. Thus, there is an imperative need for a diagnostic tool superior to ABR to accurately detect subclinical bleedings or early joint damages so that medical providers can promptly modify the treatment approach in patients with early joint damages.

Historically, magnetic resonance imaging (MRI) has been the main imaging modality for evaluating joint conditions in patients with joint bleeding. However, it has various setbacks, such as high costs; need for appointments, causing a delay in making treatment decisions; need for sedating pediatric patients; and unsuitability for assessing polyarticular conditions.

Recently, musculoskeletal ultrasound (MSKUS) has gained increasing attention due to its capability of identifying early joint damage, including joint bleeding and synovial proliferation [3,4]. MSKUS assessment of early joint damage provides findings comparable with those of MRI-based diagnostic procedures [5,6]. There are reports that MSKUS is more commonly used in actual clinical practice than MRI [7].

As MSKUS can be used to provide prompt noninvasive assessments, it is considered as a clinically valuable tool for early joint damage identification. Endorsing the perspective of the Italian Association of Hemophilia Centers, MSKUS should be used for synovitis evaluations at each clinical follow-up visit, regardless of whether joint symptoms are manifested, and in cases of detection of synovitis, the therapeutic approach should be tailored [8].

In this study, we aimed to investigate the long-term patient outcomes of utilizing periodic MSKUS as a point-of-care tool within shared decision-making processes for most patients with severe hemophilia at a single hemophilia center. To achieve this goal, we conducted the “Joint Damage Monitoring by Ultrasonography in Patients with Hemophilia in Japan” (J-DaUPHIN) study, a long-term, prospective, observational study.

## 2 Methods

### 2.1 Study design and eligibility

The J-DaUPHIN study is a prospective, noninterventional, observational study that was conducted at a hemophilia treatment center in Japan (Ogikubo Hospital, Tokyo) between March 2018 and March 2022. Data on the long-term joint outcomes of individuals with hemophilia who underwent periodic MSKUS were collected. The study protocol was approved by the local independent ethics committee (approval number: 17-D-0023) and conducted in accordance with relevant ethical guidelines. Written informed consent was obtained from all participants or their guardians before the study started.

Individuals aged ≥6 years, who had congenital moderate-to-severe hemophilia A or B (factor [F]VIII ≤ 2% or FIX ≤ 2%) and had undergone prophylaxis (regardless of factor product or regimen), were included. At the time of study enrollment, their home infusion records for at least 6 months before enrollment and for >50 exposure days (EDs) to coagulation factors for children aged <12 years, or >150 EDs for adolescents aged ≥12 years and adults, were required for enrollment. Individuals were excluded if they had acquired hemophilia; were positive for FVIII or FIX inhibitors; had end-stage arthropathy with stage IV/V of Arnold’s classification [7] in all 6 joints, consisting of both elbows, knees, and ankles, including those after joint replacement surgery, at the time of the screening; or received on-demand treatments. Based on radiographic images of the joints at screening, the joints with end-stage arthropathy classified as stage IV/V of Arnold’s classification at the time of initiating MSKUS monitoring were excluded from MSKUS joint monitoring due to difficulties in distinguishing between hemarthrosis and hydrarthrosis in these joints.

### 2.2 Monitoring of joint condition

Participants visited our hospital biannually over a 3-year period for MSKUS (LOGIQ e V2, GE HealthCare Japan) examinations of the elbows, knees, and ankles. These visits were systematically labeled from visit 1 (the initial consultation) to visit 7 (36 months after the initiation of MSKUS). A designated trained examiner, who had received direct training from the hemophilia early arthropathy detection with ultrasound (HEAD-US) group [9] in 2017, consistently managed these evaluations using MSKUS, minimizing interexaminer variability. After examination using MSKUS, the medical providers communicated the findings to the participants as part of shared decision making. When deemed necessary, the medical provider provided guidance about modifications to prophylaxis, practical advice such as the timing of the prophylaxis based on participants’ lifestyle habits, educational interventions for better adherence, recommendations for rehabilitation, and advice regarding physical activities. However, given the observational nature of the study, the detailed data collected were limited to the factor products used for prophylaxis, dosages, frequencies, product names, adherence levels, and the ratio of actual prophylactic injections administered compared with the predetermined frequency agreed upon by healthcare providers and patients. The specific medical advice provided in clinical practice was individualized for each patient through various tailored approaches, without adhering to a fixed algorithm.

MSKUS images were assessed blindly by another independent orthopedic surgeon with 35 years of experience in medical practice for hemophilia. This surgeon also received specific training from the HEAD-US group. The joint condition was classified by the severity of synovitis, cartilage damage, and bone degeneration, according to the HEAD-US scoring protocol [9], the primary scoring method for hemophilia in Japan in 2018. Although the protocol’s suitability for pediatric assessments was not yet well established, its use was deemed provisional due to the absence of other pediatric-focused scoring methods.

The Hemophilia Joint Health Score (HJHS) version 2.1 [10,11], where a high value indicates advanced arthropathy, was determined independently by either the same medical provider or a physiotherapist who had been trained by an instructional video.

Based on self-reports of home infusions (reported via patient notebook or mobile application), the number of prophylactic injections and injections for spontaneous or traumatic bleeding events during the last 6 months before the visit were independently counted by other study staff. Spontaneous annual joint bleeding rate (sAjBR) represents bleeding counts per year; it was calculated from the number of bleeding events recorded over the course of 6 months before the visit.

### 2.3 Definition of subclinical synovitis

There is no specific definition for subclinical synovitis. Therefore, we used definitions based on the sAjBR and HJHS. Based on the sAjBR, cases of synovitis detected by ultrasound but without bleeding events requiring treatment (including no “self-treatment for bleeding”) within the last 6 months were considered subclinical synovitis. Based on the HJHS, cases of synovitis detected by ultrasound but with the HJHS examination showing no joint swelling or pain were considered subclinical synovitis.

### 2.4 Statistical analyses

During the study period, participants who were lost to follow-up, who discontinued prophylaxis, who were enrolled in other clinical trials, who withdrew their consent, or whose injection logs were confirmed to be ambiguous were excluded from the statistical analyses.

Participants’ demographic and baseline characteristics, HEAD-US score, HJHS, and sAjBR at every 6-month visit were analyzed using descriptive statistics. The numbers of adjustments for prophylaxis at every visit were counted, and each adjustment was categorized. Frequencies and proportions (%) were calculated for categorical or ordinal variables. Statistical analysis was performed using the Wilcoxon signed-rank test with a significance level of .05 and 2-sided 95% CI to compare the joint scores at each visit with those at visit 1. To evaluate the effect sizes between visits, we calculated the standardized mean differences (SMDs) for each pair of visits. The SMD was computed using the formula: Δ*M*/σΔ. Δ*M* is the difference in mean scores between 2 visits, and σΔ is the SD of the change in scores between those visits. Multivariate logistic regression analyses were conducted for the elbow, knee, and ankle to examine factors associated with improvement of one point or greater in the HEAD-US total score between visits 1 and 7. These factors include age, body mass index (BMI), hemophilia A/B, history of inhibitors, number of preplanned prophylaxis, number of injections for bleeding events, number of actual prophylactic injections, HJHS, and adherence to preplanned prophylaxis for 6 months (<80%, 80%-100%, 100%-120%, and ≥120%) collected at visit 1 and sAjBR and number of adjustments for prophylaxis recorded from visits 1 to 7 throughout the study period. Statistical analyses were performed using SAS software (version 9.4; SAS Institute).

## 3 Results

### 3.1 Demographic and baseline characteristics

**Figure 1:**
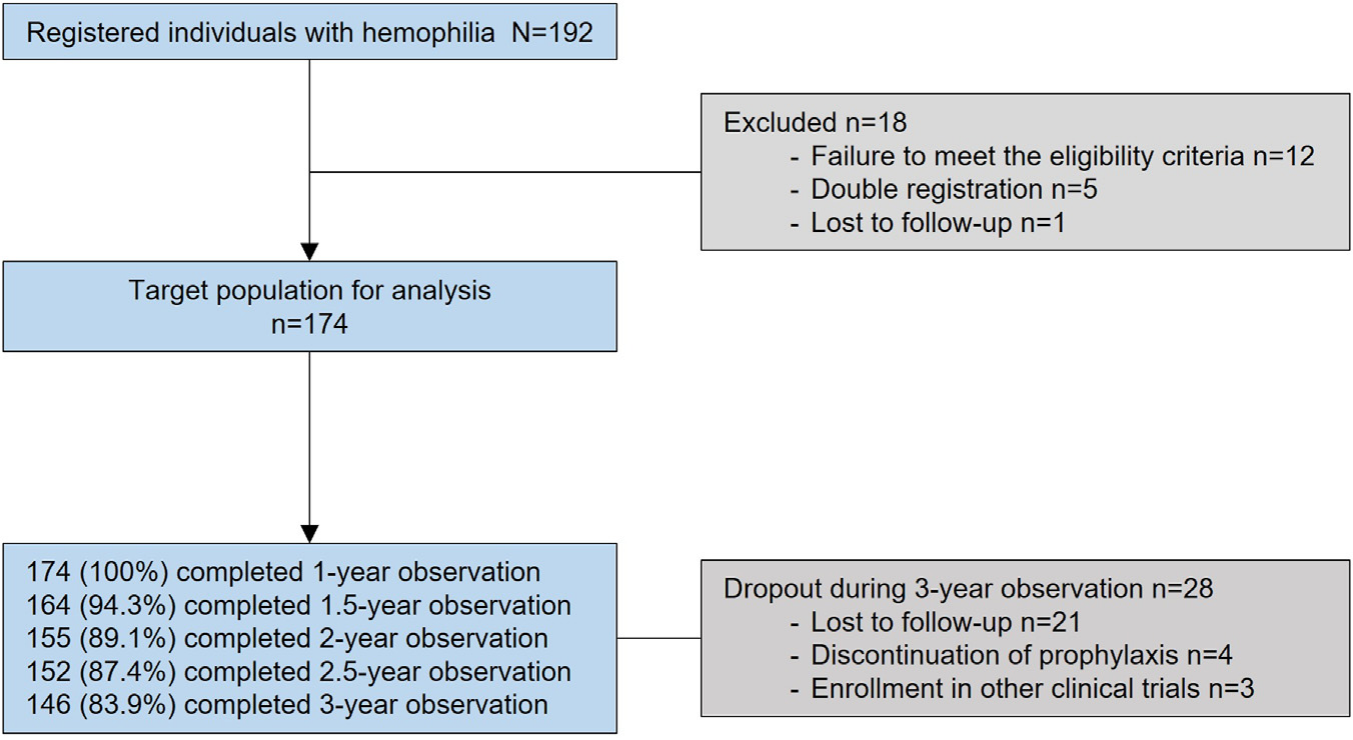
Flow diagram of the study population. The target population included all participants with hemophilia who met the eligibility criteria and completed at least 1 year of observation.

Of the 192 initially enrolled individuals with hemophilia, 174 who completed at least 1 year of observation were included as the target population, and 18 were excluded within the first year due to various reasons (Figure 1). All participants in this study were of Japanese ancestry. Of the target population of 174 participants with hemophilia, 146 (83.9%) were followed up through the entire 3-year study duration, and 28 withdrew from the study for various reasons such as loss to follow-up, mainly due to the COVID-19 pandemic (21 participants), discontinuation of prophylaxis (4 participants), and enrollment in a different clinical trial (3 participants).

The target population comprised 174 participants with hemophilia and had a median age of 34 years (range, 6-72). The age distribution was comparable across the entire population with hemophilia A and B (Table). At enrollment, 54.0% of the target population were using extended half-life products (EHL), 44.8% were using standard half-life products (SHL), and 1.1% opted for nonfactor therapy (NF). Details of the dose and dosing frequency of the prophylactic treatment are presented in Supplementary Table S1. Over the 3-year study period, 682 joints (65.3% of 1044 joints) of all 174 participants, including 238 elbows (68.4% of 348 elbows), 293 knees (84.2% of 348 knees), and 151 ankles (43.3% of 348 ankles) were scanned using MSKUS, and a total of 3582 MSKUS images were gathered.

**Table:** Demographic and baseline characteristics.

How about characteristic?	Total	Hemophilia	
		A	B
No. of participants (%)	174 (100)	144 (82.8)	30 (17.2)
Age (y), median (range)	34 (6-72)	34 (6-72)	33.5 (8-60)
Age distribution (y), *n* (%)			
6-9	9 (5.2)	8 (5.6)	1 (3.3)
10-19	24 (13.8)	21 (14.6)	3 (10.0)
20-29	36 (20.7)	28 (19.4)	8 (26.7)
30-39	41 (23.6)	34 (23.6)	7 (23.3)
40-49	45 (25.9)	37 (25.7)	8 (26.7)
≥50	19 (10.9)	16 (11.1)	3 (10.0)
BMI (kg/m^2^),^a^ mean (SD)	22.4 (4.1)	22.3 (3.9)	22.5 (4.6)
Therapeutic products at enrollment, *n* (%)			
SHL	78 (44.8)	75 (52.1)	3 (10.0)
EHL	94 (54.0)	67 (46.5)	27 (90.0)
NF	2 (1.1)	2 (1.4)	0 (0)
No. of joints scanned (%)^b^			
Total	682/1044 (65.3)	553/864 (64.0)	129/180 (71.7)
Elbow	238/348 (68.4)	192/288 (66.7)	46/60 (76.7)
Knee	293/348 (84.2)	240/288 (83.3)	53/60 (88.3)
Ankle	151/348 (43.4)	121/288 (42.0)	30/60 (50.0)
Total no. of MSKUS images over 3 y, *n*			
Total	3582	2878	704
Elbow	1260	1006	254
Knee	1532	1244	288
Ankle	790	628	162

All participants were Japanese.

BMI, body mass index; EHL, extended half-life product; MSKUS, musculoskeletal ultrasound; NF, nonfactor therapy; SHL, standard half-life product.

a Due to missing data of 1 participant with hemophilia A, the BMI was calculated with *n* = 173 for total and *n* = 143 for hemophilia A.

b Number of joints scanned using MSKUS. The percentage of scanned joints for total number of joints (right and left elbows, knees, and ankles) of all participants with hemophilia.

### 3.2 Alterations in prophylaxis strategies and adherence patterns

**Figure 2:**
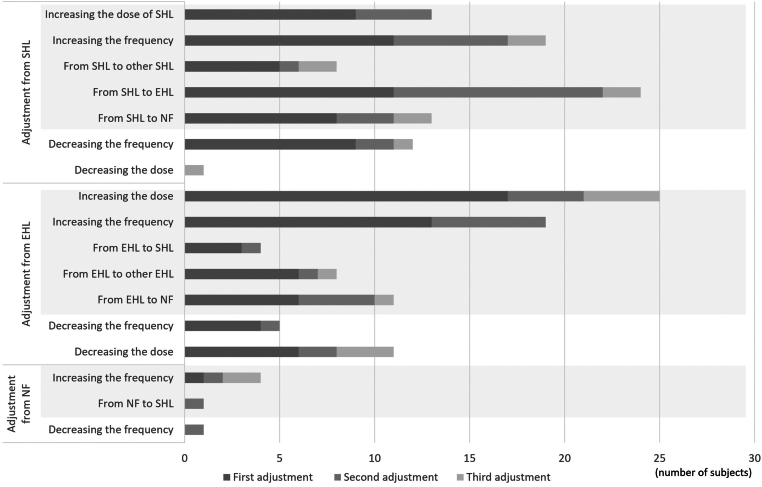
Details of adjustments for prophylaxis. The number of participants who had adjustments made to their prophylaxis regimen is shown, with some participants counted more than once if they had multiple adjustments. The darkest bar represents the number of participants who had their first adjustment, the gray bar indicates those who had a second adjustment, and the lightest bar indicates those who had a third adjustment during the follow-up period. Bars with a colored background depict adjustments such as increased dosing or switching to products with different half-lives/mechanisms of action. Dosage changes due solely to weight gain, particularly in pediatric patients, were not counted as adjustments. EHL, extended half-life product; NF, nonfactor therapy; SHL, standard half-life product.

Modifications to the prophylaxis regimen were observed throughout the 3-year study period. Among 140 participants with synovitis at any point, 97 (69.3%) took proactive measures by adjusting their prophylaxis regimen at least once: 52 (53.6%) adjusted their prophylaxis once, 29 (29.9%) adjusted it twice, and 16 (16.5%) adjusted it 3 or more times (Figure 2).

As for details of adjustments for prophylaxis in 174 participants of the target population, the first adjustment for prophylaxis was observed in 55 treated with EHL, 53 treated with SHL, and 1 treated with NF (Figure 2). The most common adjustment in EHL was an increase in dose (17 participants) and frequency (13 participants), whereas that in SHL was an increase in frequency (11 participants) and a switch to EHL (11 participants).

With regard to making the second adjustment, 19 participants treated with EHL readjusted their use of EHL, and 6 of them chose to increase the dosing frequency. For participants treated with SHL, 27 of them readjusted their use of SHL and 11 switched to EHL. Three participants treated with NF underwent the second adjustment, of whom 1 chose to increase the dose frequency.

A total of 21 participants readjusted their prophylaxis at least 3 times (Figure 2).

In addition to intensification of treatment, notable adjustments for prophylaxis included reductions in dosage or dosing frequency.

**Figure 3:**
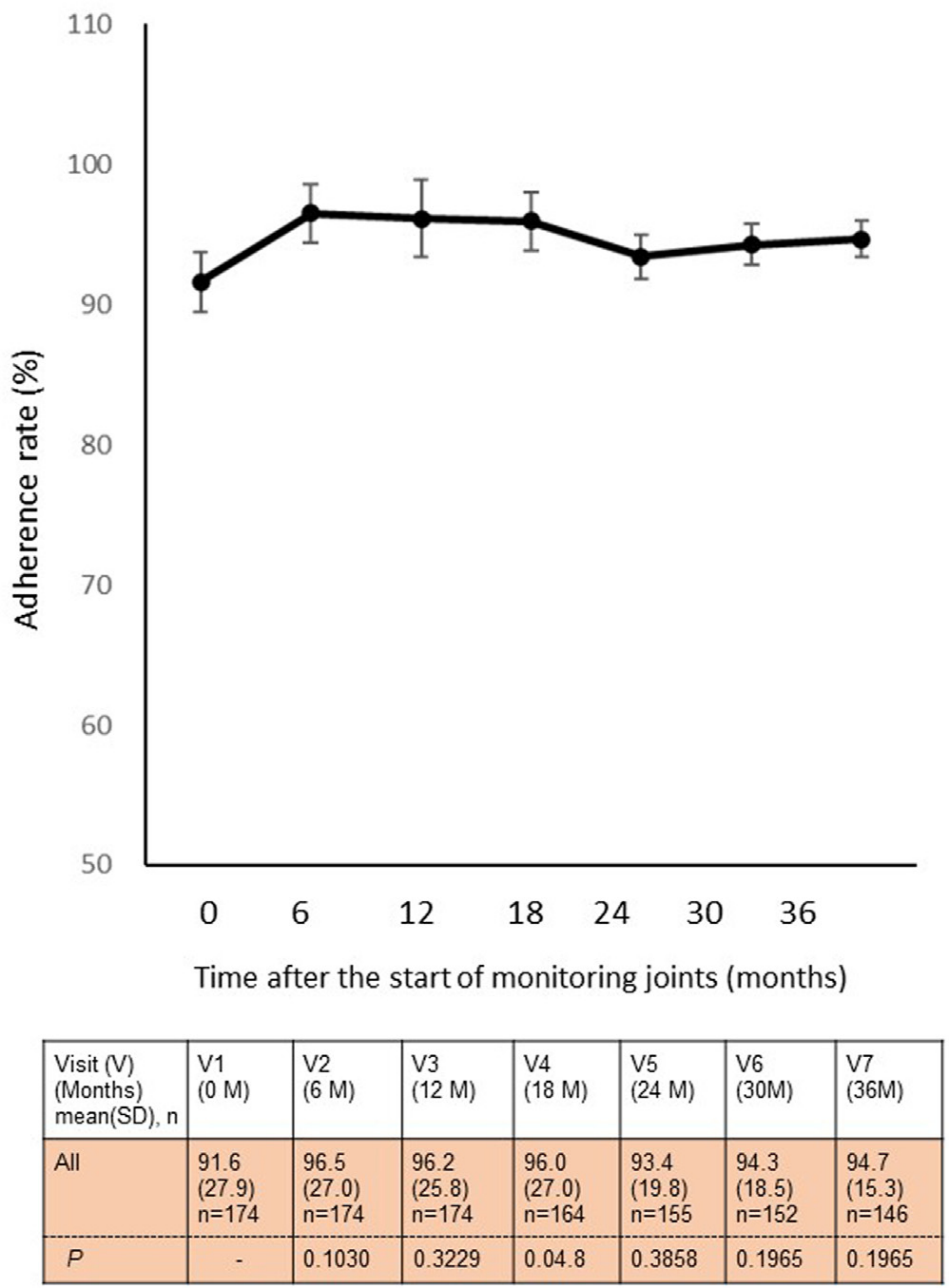
Trends in adherence to prophylaxis regimen. This figure outlines the trends by depicting the ratio of prophylaxis doses prescribed by patients and healthcare providers to the actual number of prophylaxis doses administered across each visit. The table header corresponds to time-point labels for graphs. *n*, number of participants with hemophilia.

Furthermore, Figure 3 illustrates the average adherence to the prophylaxis regimen. High adherence, at 91.6%, was already observed from visit 1, and overall adherence slightly improved over the 3-year study period, although the difference was not statistically significant.

### 3.3 Improvements in joint scores

#### 3.3.1 Improvements in the HEAD-US scores

**Figure 4:**
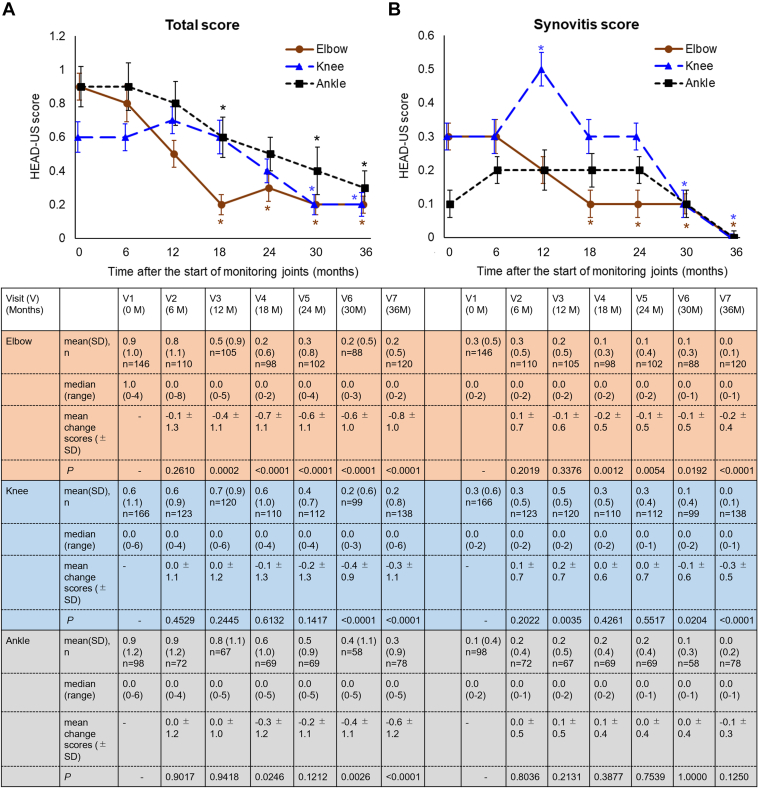
Improvement in the HEAD-US score obtained by MSKUS joint monitoring over 3 years. The HEAD-US scores of the right and left joints (elbow, knee, and ankle) were collected, and the higher score of each participant was used to calculate descriptive statistics. (A) HEAD-US total score combining HEAD-US synovitis, cartilage, and bone scores for the elbow, knee, and ankle. (B) HEAD-US synovitis score. The table header corresponds to time-point labels for graphs. Error bars represent SEs. ∗ represents significant differences with *P* < .05, as determined by the Wilcoxon signed-rank test. HEAD-US, hemophilia early arthropathy detection with ultrasound; MSKUS, musculoskeletal ultrasound; *n*, number of participants with hemophilia.

The HEAD-US total and synovitis scores of the elbow, knee, and ankle over 36 months of the study period are shown for the target population with hemophilia (Figure 4A, B). Thirty-six months after the initiation of MSKUS (visit 7), the HEAD-US total score had significantly improved in the elbow (*P* = .0002 at visit 3, 12 months after the initiation of MSKUS), knee (*P* < .0001 at visit 6, 30 months after the initiation of MSKUS), and ankle (*P* = .003 at visit 6, 30 months after the initiation of MSKUS; Figure 4A). The HEAD-US synovitis scores of the elbow and knee significantly improved at visit 7 (all *P* < .0001; Figure 4B). The effect sizes *d* for the changes from visit 1 to visit 7 were as follows: the elbow total was at 0.861, the elbow synovitis was at 0.796, the knee total was at 0.411, the knee synovitis was at 0.669, the ankle total was at 0.554, and the ankle synovitis was at 0.304. In particular, the HEAD-US synovitis score of the elbows began to significantly improve from visit 4, 18 months after the initiation of MSKUS (*P* = .001), and the improvement continued until visit 7 (for visits 5, 6, and 7, *P* = .005, *P* = .019, and *P* < .0001, respectively). Meanwhile, the HEAD-US synovitis score of the knee began to significantly improve from visit 6, 30 months after the initiation of MSKUS (for visits 6 and 7, *P* = .020 and *P* < .0001, respectively). However, the HEAD-US synovitis scores of the ankles were initially low and did not significantly change throughout the 3-year study period. Furthermore, the HEAD-US cartilage and bone scores are shown in Supplementary Figure. The cartilage score showed a significant improvement.

Multivariate analysis to identify the factors associated with improvement in the HEAD-US total score for each joint revealed that a BMI of ≥25 kg/m^2^ was significantly related to an improved HEAD-US total score of the elbow, with an odds ratio of 3.76 (95% CI, 1.34–10.55, *P* = .012; Supplementary Table S2).

#### 3.3.2 Reduction in the sAjBR and HJHS

**Figure 5:**
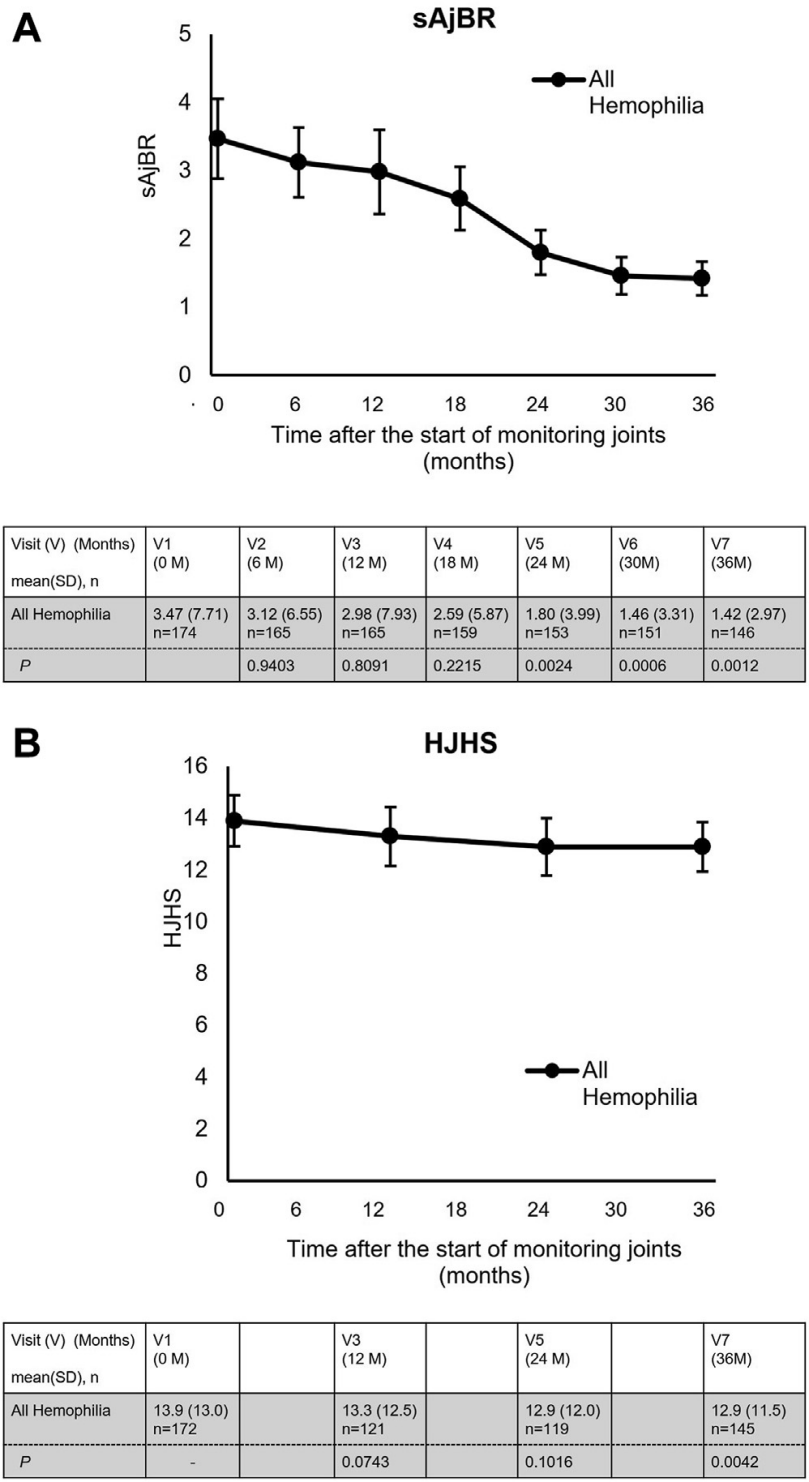
Improvement in the sAjBR and HJHS over 3 years. Trend in (A) sAjBR and (B) HJHS over 3 years. Table column headers reflect time-point labels for graphs. Error bars represent SEs. ∗ represents significant differences with *P* < .05, as determined by the Wilcoxon signed-rank test. HJHS, Hemophilia Joint Health Score; *n*, number of participants with hemophilia; sAjBR, spontaneous annual joint bleeding rate.

In all participants with hemophilia A or B, the sAjBR significantly decreased from visit 5, 24 months after the initiation of MSKUS (*P* = .002; Figure 5A). Over the 3-year study period, the mean sAjBR decreased from 3.47 to 1.42 in all participants with hemophilia A or B. Intriguingly, the median sAjBR consistently remained at 0.0. The HJHS significantly improved at visit 7 (*P* = .004; Figure 5B) as well.

#### 3.3.3 Detection of subclinical synovitis using MSKUS

**Figure 6:**
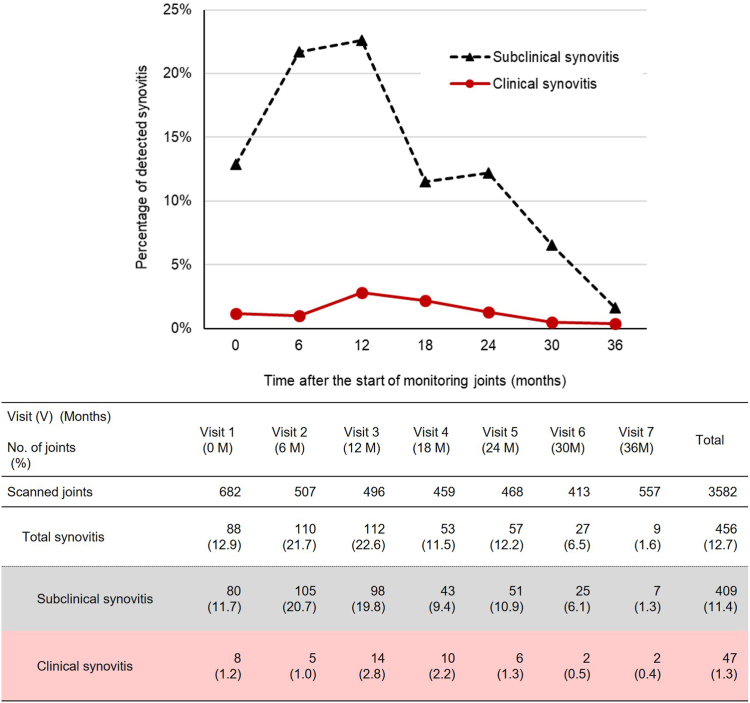
Decreased percentage of synovitis detected using MSKUS over 3 years. The numbers of scanned joints and synovitis joints detected using MSKUS are shown between visits 1 and 7. Subclinical synovitis is synovitis with no bleeding events for the last 6 months before the detection of synovitis, whereas clinical synovitis is synovitis with bleeding events for the last 6 months before the detection of synovitis. Values in brackets indicate the percentage of synovitis joints among the scanned joints. MSKUS, musculoskeletal ultrasound.

Of the 3582 joints scanned using MSKUS, synovitis with at least 1 point on HEAD-US was observed in 456 joints (12.7%), including 136 elbows, 250 knees, and 70 ankles, in 140 of the 174 (80.5%) participants. Based on the sAjBR, bleeding events in the last 6 months before detection of synovitis using MSKUS had only been observed in 47 of the 456 synovitis cases; the remaining 409 cases had no history of bleeding event (Figure 6). The percentage of total synovitis detected using MSKUS decreased gradually from 12.9% at visit 1 to 1.6% at visit 7. The percentage of subclinical synovitis with no bleeding records and clinical synovitis with bleeding records also decreased from 11.7% to 1.3% and from 1.2% to 0.4%, respectively, between visits 1 and 7. Based on the HJHS, of the 398 synovitis joints, only 11 (2.8%) had swelling, 12 (3.0%) had pain, and 3 (0.8%) had both swelling and pain.

The age distribution of synovitis was analyzed to identify the age group most strongly affected by subclinical synovitis (Supplementary Table S3). The incidence of synovitis increased with age. Of note, although there were a few synovitis cases in the 6-to-9-year-old group, they were all subclinical.

## 4 Discussion

During the 3-year observational period, tailored prophylaxis, primarily based on adjustments resulting from shared decision making, saw further enhancement. This approach not only gradually improved adherence to prophylaxis but also led to significant improvements in HEAD-US scores, a reduction in patient-reported bleeding, and improvement in HJHS.

Rodriguez-Merchan [12] notes in his editorial, US findings can facilitate the appropriate adjustment of prophylaxis regimens (“I believe”). Endorsements by esteemed institutions like the Italian Association of Hemophilia, European research groups, as well as the World Federation of Hemophilia, underscore the vital role of MSKUS in identifying synovitis, thus enabling clinicians to personalize prophylaxis [8,13,14]. Indeed, there are consecutive reports indicating that MSKUS is being utilized for treatment individualization [[15], [16], [17]].

In our cohort, 69.3% adjusted their prophylaxis in response to findings of MSKUS, which was reflected in improved HEAD-US scores. Even those who continued with their original regimen observed improvement in their scores of HEAD-US, which may be attributable to increased adherence to their treatment regimen, spurred by educational interventions and in-depth explanations from healthcare professionals. Various therapeutic interventions, such as increased focus on joint health, physiotherapy, and anti-inflammatory management, also contributed to these results.

Based on MSKUS images, some participants managed to lessen the dose or the dosing frequency. Such adjustments not only cater to individual patient needs but also contribute to healthcare cost savings by reducing medication consumption. Within this study, a wide variety of shared decisions were made, making it challenging to present a clear treatment algorithm, but this is precisely what constitutes tailored prophylaxis. Furthermore, in the United States, there seemed to be a tendency to use MSKUS more for physiotherapeutic management than for medical management, likely influenced by differences in insurance and healthcare costs [15].

Given that 11.7% of synovitis detections were subclinical, this study underscores the crucial importance of tools like MSKUS in uncovering these hidden signs of joint damage. Furthermore, as all 13 children aged <10 years experienced subclinical synovitis, intervals longer than the 6 months used in this study may not be recommended for MSKUS examination. However, it is difficult to conclude on the recommended examination interval for adults based solely on this study. Moreover, the existence of subclinical synovitis itself might be questioned. Of course, the results from the Joint outcome study are emblematic [1], but recent data, including those from a report by van Leeuwen et al. [18], revealed that MRI detected subclinical synovitis in 16% of joints in patients aged 16 to 33 years with no lifetime history of bleeding in those joints [19]. Similar findings were reported in ultrasound examinations, where 12.7% of joints with no lifetime bleeding history had a HEAD-US score greater than 1 [20]. The existence of subclinical synovitis can thus be considered confirmed. The definition of subclinical synovitis varies, with the 2 aforementioned examples involving joints with no lifetime bleeding history, though tracking a lifetime history of bleeding through relocation or record inaccuracies is challenging. Van Bergen et al. [21] proposed their own definition, and comparisons, such as with swelling in HJHS, vary, with the proportion differing by population and definition, as revealed in meta-analyses [22]. The definition of subclinical synovitis in this study was not based on a lifetime of bleeding but rather on no recollection of bleeding in the past 6 months, despite findings in MSKUS aligning with the subclinical rates in other studies. Ultimately, the importance does not lie on the definition or proportion but on how these results are utilized clinically. In our study, the incidence of subclinical synovitis decreased to 1.3% over the 3-year MSKUS joint monitoring period.

As a result of exclusion of joints with end-stage arthropathy, the number of study joints varied (enrollment numbers: 68.4% of 348 elbows, 84.2% of 348 knees, 43.3% of 348 ankles). This reflects that many participants had end-stage arthropathy in their ankles.

The HEAD-US score of all 3 joints (elbow, knee, and ankle) improved significantly; in particular, an improvement in the HEAD-US score of the elbow was observed early after initiation of MSKUS, possibly because the elbow joint does not bear weight. The frequency of bleeding and synovitis in the knee and ankle, which are weight-bearing joints, can be more affected by physical activity than that in the elbows. We did not monitor physical activity, which could be considered one of the limitations of this study, because the proportion of end-stage arthropathy cases in a patient sample can vary with physical activity, physical activity levels can change over time, and information collected through questionnaires is prone to cognitive biases. However, we assumed that the joint condition in the elbow is less associated with physical activity than that in other assessed joints, which is reflected in our observation that the HEAD-US score in the elbow improved promptly after initiation of MSKUS.

We found that a high BMI (≥25 kg/m^2^) was significantly related to improvements in the HEAD-US total score of the elbow from visits 1 to 7. Although the mechanism behind this association is unclear, participants with a higher BMI were more likely to have a HEAD-US score of ≥1 of the elbows at visit 1, probably caused by strain on the elbow. Moreover, long-term periodic MSKUS might have improved the HEAD-US score of the elbow that is not involved in physical activity as much as the other assessed joints.

Despite the irreparability of damaged natural cartilage, the HEAD-US cartilage score improved, suggesting that the score may not accurately reflect the actual condition. Hence, in cases where MSKUS detects cartilage damage along with synovitis or bleeding, it is imperative to maintain regular follow-up examinations.

At the commencement of our study, the applicability of HEAD-US to pediatric populations remained unclear and was deemed provisional due to the absence of other pediatric-focused scoring methods. However, recent reports have verified its reliability in comparison with MRI for children aged ≥6 years [5]. Consequently, the HEAD-US evaluations in this study can be considered as reliable data. Children aged 6 to 9 years displayed the highest incidence of subclinical synovitis. Although the overall number of children in this age group was relatively small compared with the entire study population, this result underlines the necessity for regular MSKUS monitoring for children with hemophilia. Boccalandro et al. [23] also argue that utilizing ultrasound to assess infants before the initiation of primary prophylaxis can prevent subclinical bleeding. This approach leads to an enhanced understanding of early prophylaxis and promotes shared decision making [23].

Some limitations of this study include the absence of a comparison with participants without hemophilia or a group that did not undergo ultrasound examinations. A control group could not be included owing to the study’s observational nature; therefore, controlled trials are warranted in the future. Alternatively, establishing a control group comprising patients with hemophilia who do not undergo routine MSKUS monitoring might have been judicious. However, abstaining from utilizing available monitoring technology poses a notable ethical dilemma. It should be noted that asymptomatic mild synovitis may be observed in healthy young individuals with a certain level of physical activity [24]. However, this study did not collect data on physical activity levels, which is a limitation. In future studies, incorporating objective assessments of physical activity would be crucial. Additionally, recent reports have described the concept of inactive synovitis [21]. As this study utilized HEAD-US without power Doppler, we were unable to evaluate inactive synovitis. Another limitation was excluding joints with end-stage arthropathy due to concerns about distinguishing between hydrarthrosis and hemarthrosis with MSKUS. Because of concerns that overestimating hydrarthrosis as hemarthrosis clinically could cause excessive medical interventions and increased healthcare costs, we excluded end-stage arthropathy from monitoring joint conditions using MSKUS. In addition, this study did not validate findings with MRI. Historically, MRI has been the gold standard for identifying joint bleeding and synovitis in hemophilia. However, MRI comes with its own set of limitations, especially in distinguishing blood from nonblood fluids [25]. Furthermore, while hemosiderin deposition is regarded as the gold standard for identifying bleeding, MRI is necessary to detect hemosiderin and confirm acute bleeding. Yet, no literature evaluates the presence of hemosiderin deposition in chronic synovitis. Comparisons between MSKUS and MRI have been documented [5,6]. Therefore, given that the study population comprised patients with hemophilia and considering the pathology within the joint, we believe that findings detected on ultrasound likely indicate intra-articular bleeding or synovitis. Moreover, we excluded patients who were not receiving prophylactic treatment. Patients on on-demand treatment could potentially benefit from MSKUS findings by being motivated to initiate prophylaxis. Future studies should also investigate the impact of incorporating MSKUS for this subgroup of patients to provide a more comprehensive evaluation of the utility of MSKUS across the spectrum of hemophilia management approaches. Finally, in this study, we did not collect information on physiotherapy, anti-inflammatory management, or clotting factor levels and thus could not evaluate their impact on the outcomes. This information is being collected in an ongoing research.

Medications for hemophilia will continue to evolve. Considering the increasing number of patients receiving nonfactor therapy or gene therapy, this study is ongoing as a prospective research over a span of 10 years.

## 5 Conclusions

In conclusion, our study underscores the imperativeness of early detection and intervention, which are paramount for augmenting the quality of life for individuals with hemophilia, thereby reinforcing the necessity of incorporating such diagnostic modalities in routine clinical practice.

## Supplementary material

Supplemental Tables

Supplementary Figure Cartilage and bone scores of the HEAD-US score The HEAD-US scores of the right and left joints (elbow, knee, and ankle) were collected, and the higher score of each participant was used to calculate the descriptive statistics. (A) The HEAD-US cartilage scores for the elbow, knee, and ankle. (B) The HEAD-US bone score. The table header corresponds to time-point labels for the graphs. Error bars represent standard errors. ∗Represents significant differences with *P* < .05, as determined by the Wilcoxon signed-rank test. HEAD-US, hemophilia early arthropathy detection with ultrasound; MSKUS, musculoskeletal ultrasound; n, number of participants with hemophilia; SD, standard deviation.
