# Supplementary material for: Integrating musculoskeletal ultrasound as a shared decision-making tool in hemophilia care: observations from a 3-year study
Source: Res Pract Thromb Haemost. 2024 Jul 14;8(5):102511. doi: 10.1016/j.rpth.2024.102511 (PMC11347854; doi:10.1016/j.rpth.2024.102511)
Supplement: Supplemental Tables [file mmc1.docx]

Supplementary Table S1. Details of Prophylactic Treatment Protocols at Registration

|  |  | Products for Hemophilia A | | | | | | | | | | | Products for Hemophilia B | | | | | | | | | | | | |  |  |  |  |  |  |
| --- | --- | --- | --- | --- | --- | --- | --- | --- | --- | --- | --- | --- | --- | --- | --- | --- | --- | --- | --- | --- | --- | --- | --- | --- | --- | --- | --- | --- | --- | --- | --- |
|  |  |  |  |  |  |  |  |  |  |  |  |  |  |  |  |  |  |  |  |  |  |  |  |  |  |  | | | |  |  |
|  |  | EHL-1 | | EHL-2 | | SHL-1 | | SHL-2 | | Non-factor | | Other | EHL-1 | | EHL-2 | | | SHL-1 | PD | | | Other | | |  | | | |  |  |  |
| Number of cases | | 44 | | 29 | | 29 | | 30 | | 2 | | 21 | 13 | | 17 | | | 1 | 2 | | | 0 | | |  | | | |  |  |  |
| Dosage ^*1^ | |  | |  | |  | |  | |  | |  |  | |  | | |  |  | | |  | | |  | | | |  |  |  |
|  | Mean ± Standard Deviation | 2744.3 ± 757.7 | | 2275.9 ± 701.9 | | 1793.1 ± 644.6 | | 2166.7 ± 530.7 | | 152.5 ± 38.9 | | 2381.0 ± 740.0 | 3461.5 ± 967.4 | | 3411.8 ± 1003.7 | | | 2000.0 ± - | 4000.0 ± 2828.4 | | |  | | |  | | | |  |  |  |
|  | Median | 3000.0 | | 2000.0 | | 2000.0 | | 2000.0 | | 152.5 | | 3000.0 | 4000.0 | | 3000.0 | | | 2000.0 | 4000.0 | | |  | | |  | | | |  |  |  |
|  | Minimum - Maximum | 750 ～ 5000 | | 1000 ～ 4000 | | 750 ～ 3000 | | 1000 ～ 3000 | | 125 ～ 180 | | 1000 ～ 3000 | 2000 ～ 5000 | | 2000 ～ 6000 | | | 2000 ～ 2000 | 2000 ～ 6000 | | |  | | |  | | | |  |  |  |
| Dosing Interval | Every Other Day | 0 (0.0%) | | 0 (0.0%) | | 5 (17.2%) | | 3 (10.0%) | | 0 (0.0%) | | 1 (4.8%) | 0 (0.0%) | | 0 (0.0%) | | | 0 (0.0%) | 0 (0.0%) | | |  | | |  | | | |  |  |  |
|  | Once Weekly | 18 (40.9%) | | 6 (20.7%) | | 1 (3.4%) | | 3 (10.0%) | | 1 (50.0%) | | 0 (0.0%) | 3 (23.1%) | | 16 (94.1%) | | | 0 (0.0%) | 0 (0.0%) | | |  | | |  | | | |  |  |  |
|  | Twice Weekly | 19 (43.2%) | | 21 (72.4%) | | 8 (27.6%) | | 10 (33.3%) | | 0 (0.0%) | | 10 (47.6%) | 0 (0.0%) | | 0 (0.0%) | | | 1 (100.0%) | 2 (100.0%) | | |  | | |  | | | |  |  |  |
|  | Tree Times Weekly | 1 (2.3%) | | 0 (0.0%) | | 13 (44.8%) | | 13 (43.3%) | | 0 (0.0%) | | 8 (38.1%) | 0 (0.0%) | | 0 (0.0%) | | | 0 (0.0%) | 0 (0.0%) | | |  | | |  | | | |  |  |  |
|  | Four times Weekly | 0 (0.0%) | | 0 (0.0%) | | 0 (0.0%) | | 0 (0.0%) | | 0 (0.0%) | | 0 (0.0%) | 0 (0.0%) | | 0 (0.0%) | | | 0 (0.0%) | 0 (0.0%) | | |  | | |  | | | |  |  |  |
|  | Five Times Weekly | 0 (0.0%) | | 0 (0.0%) | | 0 (0.0%) | | 0 (0.0%) | | 0 (0.0%) | | 0 (0.0%) | 0 (0.0%) | | 0 (0.0%) | | | 0 (0.0%) | 0 (0.0%) | | |  | | |  | | | |  |  |  |
|  | Six Times Weekly | 0 (0.0%) | | 0 (0.0%) | | 0 (0.0%) | | 0 (0.0%) | | 0 (0.0%) | | 0 (0.0%) | 0 (0.0%) | | 0 (0.0%) | | | 0 (0.0%) | 0 (0.0%) | | |  | | |  | | | |  |  |  |
|  | Seven Times Weekly | 0 (0.0%) | | 0 (0.0%) | | 0 (0.0%) | | 0 (0.0%) | | 0 (0.0%) | | 0 (0.0%) | 0 (0.0%) | | 0 (0.0%) | | | 0 (0.0%) | 0 (0.0%) | | |  | | |  | | | |  |  |  |
|  | Once Every 2 Weeks | 3 (6.8%) | | 0 (0.0%) | | 0 (0.0%) | | 0 (0.0%) | | 0 (0.0%) | | 0 (0.0%) | 7 (53.8%) | | 1 (5.9%) | | | 0 (0.0%) | 0 (0.0%) | | |  | | |  | | | |  |  |  |
|  | Once Every 4 weeks | 0 (0.0%) | | 0 (0.0%) | | 0 (0.0%) | | 0 (0.0%) | | 1 (50.0%) | | 0 (0.0%) | 3 (23.1%) | | 0 (0.0%) | | | 0 (0.0%) | 0 (0.0%) | | |  | | |  | | | |  |  |  |
|  | Other | 3 (6.8%) | | 2 (6.9%) | | 2 (6.9%) | | 1 (3.3%) | | 0 (0.0%) | | 2 (9.5%) | 0 (0.0%) | | 0 (0.0%) | | | 0 (0.0%) | 0 (0.0%) | | |  | | |  | | | |  |  |  |
|  |  |  |  | |  | |  | |  | |  | |  |  | |  | | |  | | | |  | |  | | | |  |  |  |
| *1: Dosage for Non-factor is in "mg", others are in "IU" | | | | | | | | |  | |  | |  |  | |  | | |  | | | |  | |  | | | |  |  |  |
| *Targets drugs administered from 6 months prior to registration date to registration date. Counts are duplicated if regimen was switched. | | | | | | | | | | | | | | | | | | | | | | | | | | | |  | | | |
| *If start date is unknown, handled as follows: If year unknown: 2013, if month unknown: January, if day unknown: 1st | | | | | | | | | | | | | | | | | | | | |  | | |  | | |  | | | |  |
| EHL: Extended Half-life products, SHL: Standard Half-life Products, PD: plasma-derived product | | | | | | | | | | | | | | | | |  | | |  | | | |  | | |  | | | |  |

Supplementary Table S2. Univariate and multivariate logistic regression analyses of factors contributing to improved HEAD-US total score from Visits 1 to 7.

| Variable | | | No. of participants | No. of responders (%) | Univariate analysis |  | Multivariate analysis |  |
| --- | --- | --- | --- | --- | --- | --- | --- | --- |
|  | | |  |  | Odds ratio (95% CI) | *P* | Odds ratio (95% CI) | *P* |
| Elbow | | |  |  |  |  |  |  |
| Age (years) | | | 120 | 59 (49.2) | 1.01 (0.99–1.04) | .2795 |  |  |
|  | | < 13 (ref) | 10 | 2 (20.0) |  |  |  |  |
|  | | ≥ 13 | 110 | 57 (51.8) | 4.30 (0.87–21.18) | .0728 | 2.07 (0.37–11.58) | .4084 |
| BMI (kg/m^2^) | | < 25 (ref) | 96 | 41 (42.7) |  |  |  |  |
|  | | ≥ 25 | 24 | 18 (75.0) | 4.02 (1.47–11.03) | .0068 | 3.76 (1.34–10.55) | .0118 |
| Diagnosis | | Hemophilia A (ref) | 100 | 48 (48.0) |  |  |  |  |
|  | | Hemophilia B | 20 | 11 (55.0) | 1.32 (0.50–3.47) | .5683 |  |  |
| History of inhibitor | | No (ref) | 113 | 56 (49.6) |  |  |  |  |
|  | | After disappearance | 7 | 3 (42.9) | 0.76 (0.16–3.57) | .7314 |  |  |
| No. of preplanned prophylaxis | | | 120 | 59 (49.2) | 0.99 (0.97–1.00) | .0921 | 1.01 (0.98–1.04) | .5155 |
| No. of injections for bleeding events | | | 75 | 36 (48.0) | 1.06 (0.99–1.13) | .1163 |  |  |
| No. of actual prophylaxis injections | | | 120 | 59 (49.2) | 0.98 (0.96–1.00) | .0232 | 0.97 (0.94–1.01) | .1317 |
| Adherence to prophylaxis | | |  |  |  |  |  |  |
|  | | < 80% (ref) | 31 | 18 (58.1) |  |  |  |  |
|  | | 80% to 100% | 42 | 20 (47.6) | 0.66 (0.26–1.67) | .3782 |  |  |
|  | | 100% to 120% | 39 | 16 (41.0) | 0.50 (0.19–1.31) | .1587 |  |  |
|  | | ≥ 120% | 8 | 5 (62.5) | 1.20 (0.24–5.96) | .8203 |  |  |
| sAjBR (total) | |  | 120 | 59 (49.2) | 1.03 (0.94–1.11) | .5530 |  |  |
| sAjBR (elbow) | |  | 120 | 59 (49.2) | 0.96 (0.66–1.41) | .8457 |  |  |
| sAjBR (knee) | |  | 120 | 59 (49.2) | 1.15 (0.86–1.54) | .3482 |  |  |
| sAjBR (ankle) | |  | 120 | 59 (49.2) | 1.03 (0.92–1.15) | .6351 |  |  |
| HJHS | |  | 118 | 58 (49.2) | 1.02 (0.99–1.06) | .1764 |  |  |
| No. of adjustment for prophylaxis | | | 120 | 59 (49.2) | 0.99 (0.74–1.32) | .9231 |  |  |
| Knee | |  |  |  |  |  |  |  |
| Age (years) | |  | 138 | 40 (29.0) | 1.03 (1.00–1.06) | .0308 | 1.03 (0.99–1.07) | .2162 |
|  | | < 13 (ref) | 10 | 0 (0.0) |  |  |  |  |
|  | | ≥ 13 | 128 | 40 (31.3) | Inf | .9750 |  |  |
| BMI (kg/m^2^) | | < 25 (ref) | 112 | 34 (30.4) |  |  |  |  |
|  | | ≥ 25 | 26 | 6 (23.1) | 0.69 (0.25–1.87) | .4629 |  |  |
| Diagnosis | | Hemophilia A (ref) | 116 | 31 (26.7) |  |  |  |  |
|  | | Hemophilia B | 22 | 9(40.9) | 1.90 (0.74–4.88) | .1832 |  |  |
| History of inhibitor | | No (ref) | 131 | 38 (29.0) |  |  |  |  |
|  | | After disappearance | 7 | 2 (28.6) | 0.98 (0.18–5.27) | .9803 |  |  |
| No. of preplanned prophylaxis | | | 138 | 40 (29.0) | 0.99 (0.97–1.00) | .1691 |  |  |
| No. of injections for bleeding events | | | 84 | 25 (29.8) | 1.02 (0.96–1.08) | .5546 |  |  |
| No. of actual prophylaxis injections | | | 138 | 40 (29.0) | 0.99 (0.97–1.00) | .1461 |  |  |
| Adherence to prophylaxis | | |  |  |  |  |  |  |
|  | | < 80% (ref) | 36 | 13 (36.1) |  |  |  |  |
|  | | 80% to 100% | 46 | 12 (26.1) | 0.62 (0.24–1.61) | .3294 |  |  |
|  | | 100% to 120% | 48 | 12 (25.0) | 0.59 (0.23–1.51) | .2724 |  |  |
|  | | ≥ 120% | 8 | 3 (37.5) | 1.06 (0.22–5.18) | .9411 |  |  |
| sAjBR (total) | |  | 138 | 40 (29.0) | 1.02 (0.94–1.11) | .6420 |  |  |
| sAjBR (elbow) | |  | 138 | 40 (29.0) | 1.19 (0.86–1.64) | .3029 |  |  |
| sAjBR (knee) | |  | 138 | 40 (29.0) | 1.11 (0.78–1.57) | .5650 |  |  |
| sAjBR (ankle) | |  | 138 | 40 (29.0) | 1.01 (0.89–1.13) | .9267 |  |  |
| HJHS | |  | 136 | 38 (27.9) | 1.03 (1.00–1.06) | .0953 | 1.01 (0.97–1.05) | .7744 |
| No. of adjustment for prophylaxis | | | 138 | 40 (29.0) | 1.01 (0.74–1.37) | .9566 |  |  |
| Ankle | |  |  |  |  |  |  |  |
| Age (years) | |  | 77 | 33 (42.9) | 1.06 (1.02–1.10) | .0052 | 1.04 (1.00–1.09) | .0436 |
|  | | < 13 (ref) | 10 | 2 (20.0) |  |  |  |  |
|  | | ≥ 13 | 67 | 31 (46.3) | 3.44 (0.68–17.44) | .1351 |  |  |
| BMI (kg/m^2^) | | < 25 (ref) | 64 | 27 (42.2) |  |  |  |  |
|  | | ≥ 25 | 13 | 6 (46.2) | 1.17 (0.35–3.89) | .7923 |  |  |
| Diagnosis | | Hemophilia A (ref) | 61 | 26 (42.6) |  |  |  |  |
|  | | Hemophilia B | 16 | 7 (43.8) | 1.05 (0.34–3.18) | .9353 |  |  |
| History of inhibitor | | No (ref) | 71 | 32 (45.1) |  |  |  |  |
|  | | After disappearance | 6 | 1 (16.7) | 0.24 (0.03–2.19) | .2080 |  |  |
| No. of preplanned prophylaxis | | | 77 | 33 (42.9) | 0.99 (0.97–1.01) | .1632 |  |  |
| No. of injections for bleeding events | | | 44 | 20 (45.5) | 1.07 (0.95–1.20) | .2877 |  |  |
| No. of actual prophylaxis injections | | | 77 | 33 (42.9) | 0.99 (0.97–1.01) | .3186 |  |  |
| Adherence to prophylaxis | | |  |  |  |  |  |  |
|  | < 80% (ref) | | 20 | 9 (45.0) |  |  |  |  |
|  | 80% to 100% | | 26 | 10 (38.5) | 0.76 (0.23–2.49) | .6555 |  |  |
|  | 100% to 120% | | 28 | 11 (39.3) | 0.79 (0.25–2.53) | .6924 |  |  |
|  | ≥ 120% | | 3 | 3 (100.0) | Inf | .9752 |  |  |
| sAjBR (total)* |  | | 77 | 33 (42.9) | 1.24 (0.97–1.58) | .0925 |  |  |
| sAjBR (elbow) |  | | 77 | 33 (42.9) | 1.64 (0.76–3.55) | .2096 |  |  |
| sAjBR (knee)* |  | | 77 | 33 (42.9) | 2.21 (1.04–4.66) | .0380 | 1.70 (0.75–3.85) | .2075 |
| sAjBR (ankle) |  | | 77 | 33 (42.9) | 1.12 (0.83–1.50) | .4655 |  |  |
| HJHS |  | | 75 | 32 (42.7) | 1.04 (0.98–1.09) | .1650 |  |  |
| No. of adjustment for prophylaxis | | | 77 | 33 (42.9) | 0.62 (0.40–0.95) | .0271 | 0.68 (0.44–1.05) | .0822 |

Multivariate logistic regression analyses for the elbow, knee, and ankle were performed for variables with *P* values of < .1 in the univariate analyses. A “responder” was defined as a participant exhibiting an improvement of 1 point or greater in the HEAD-US total score between Visits 1 and 7. Age, BMI, diagnosis, inhibitors, number of preplanned prophylaxis, number of injections for bleeding events, number of actual prophylaxis injections, adherence to prophylaxis, and HJHS were recorded at Visit 1. The sAjBR and number of adjustment for prophylaxis were assessed from Visits 1 to 7. *Univariate analyses for sAjBR (total) and for sAjBR (ankle) yielded a *P* value of < .1, and sAjBR (total) correlated to sAjBR (knee) (correlation coefficient: 0.6). We therefore used the sAjBR (knee) with a *P*-value of .0380, which was smaller than the *P* value for sAjBR (total), as a variable for the multivariate logistic regression analyses.

BMI, body mass index; CI, confidence interval; HEAD-US, Hemophilia Early Arthropathy Detection with Ultrasound; HJHS, Hemophilia Joint Health score; Inf, infinity; ref, Reference value=1; sAjBR, spontaneous Annual joint Bleeding Rate.

Supplementary Table S3. Age distribution of patients with synovitis.

| No. of joints (%) | | Scanned joints | Synovitis* | Subclinical synovitis |
| --- | --- | --- | --- | --- |
| Age, years | 6–9 | 270 | 13 (4.8) | 13/13 (100) |
|  | 10–19 | 740 | 95 (12.8) | 78/95 (82.1) |
|  | 20–29 | 897 | 108 (12.0) | 97/108 (89.8) |
|  | 30–39 | 701 | 84 (12.0) | 78/84 (92.8) |
|  | 40–49 | 726 | 111 (15.3) | 102/111 (91.9) |
|  | ≥50 | 248 | 45 (18.1) | 41/45 (91.1) |
| Total |  | 3582 | 456 (12.7) | 409/456 (89.7) |

Numbers of scanned joints using MSKUS, synovitis detected using MSKUS, and subclinical synovitis without bleeding record for the last 6 months before the detection of synovitis are listed by age group. *Values in brackets indicate the percentage of synovitis joints among the scanned joints.

MSKUS, musculoskeletal ultrasound.
