# Supplementary figures and images for: Integrating musculoskeletal ultrasound as a shared decision-making tool in hemophilia care: observations from a 3-year study
Source: Res Pract Thromb Haemost. 2024 Jul 14;8(5):102511. doi: 10.1016/j.rpth.2024.102511 (PMC11347854; doi:10.1016/j.rpth.2024.102511)

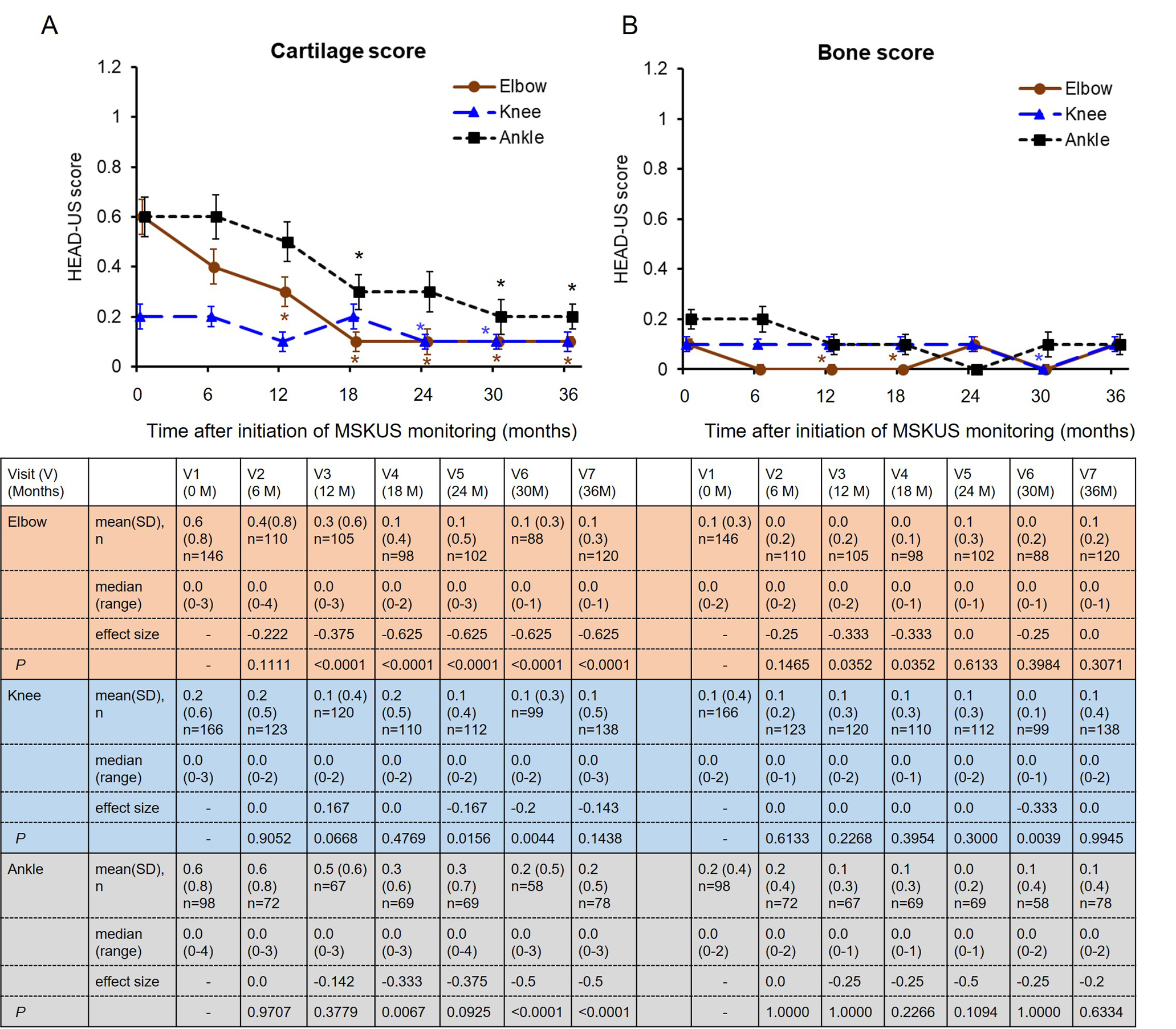

Supplement: Supplementary Figure — Cartilage and bone scores of the HEAD-US score The HEAD-US scores of the right and left joints (elbow, knee, and ankle) were collected, and the higher score of each participant was used to calculate the descriptive statistics. (A) The HEAD-US cartilage scores for the elbow, knee, and ankle. (B) The HEAD-US bone score. The table header corresponds to time-point labels for the graphs. Error bars represent standard errors. ∗Represents significant differences with P < .05, as determined by the Wilcoxon signed-rank test. HEAD-US, hemophilia early arthropathy detection with ultrasound; MSKUS, musculoskeletal ultrasound; n, number of participants with hemophilia; SD, standard deviation. [file figs1.jpg]
